# Availability of anti-rabies vaccine and rabies immunoglobulin in Indian health facilities: a nationwide cross-sectional health facility survey

**DOI:** 10.1016/j.lansea.2025.100608

**Published:** 2025-06-13

**Authors:** Navaneeth S. Krishna, Jeromie Wesley Vivian Thangaraj, Shanmugasundaram Devika, Aruna Sasi, Suganya Egambaram, D Sudha Rani, Siraj Ahmed Khan, Anitha Delli, Ashok Kumar Srivastava, Ayush Mishra, Basavaraj Shrinivasa, Chandhini Pandiyan, Devendra Gour, Debjani Ram Purakayastha, Nirmal Verma, Parul Sharma, Ravinder Kumar Soni, Sabarinatha Ramasamy, Sreelakshmi Mohandas K, Subrata Baidya, Tanveer Rehman, Vijay V. Yeldandi, Akashdeep Singh, Aswathy Sreedevi, Babasaheb V. Tandale, Chokkalingam Durairajan, Hemant Mahajan, Kamlesh Jain, Mahendra M. Reddy, Manju Toppo, Nitinkumar Valjibhai Solanki, Pramit Ghosh, Shaili Vyas, Shampa Das, Subrata Kumar Palo, Venela Prasanth, Atefh Ali, Viswanath Guru Bathin, Dinesh Kumar Sahu, G.P. Sabitha Rani, Major Madhukar, Kamran Zaman, Krishna Pandey, M Punnam Chander, Rajni Kant, Reshmi Ann Varkey, Sanghamitra Pati, Shailendra Agarwal, Srashti Panwar, Vishnu B. Menon, Raman Swathy Vaman, Anoop Velayudhan, Sam Joy, Manju Rahi, Manoj V. Murhekar

**Affiliations:** a ICMR-National Institute of Epidemiology, Chennai, Tamil Nadu, India; b ICMR-Regional Medical Research Center, Dibrugarh, Assam, India; c Himalayan Institute Medical Sciences, Swami Rama Himalayan University, Dehradun, Uttarakhand, India; d ICMR-Regional Medical Research Centre, Gorakhpur, Uttar Pradesh, India; e ICMR-National Institute of Virology, Pune, Maharashtra, India; f Gandhi Medical College, Bhopal, Madhya Pradesh, India; g ICMR-Rajendra Memorial Research Institute of Medical Sciences, Patna, Bihar, India; h Pt. Jawahar Lal Nehru Memorial Medical College, Raipur, Chhattisgarh, India; i GMERS Medical College and Hospital, Patan, Gujarat, India; j Dayanand Medical College and Hospital, Ludhiana, Punjab, India; k Amrita Institute of Medical Sciences, Amrita Vishwa Vidyapeetham, Kochi, Kerala, India; l Agartala Government Medical College & MRHRU, Tripura, India; m ICMR-Regional Medical Research Centre, Bhubaneswar, Odisha, India; n SHARE India, Hyderabad, Telangana, India; o Directorate of Health Service, Kerala, India; p ICMR, New Delhi, India; q ICMR - Vector Control Research Centre, Pondicherry, India

**Keywords:** Rabies, Vaccine, Immunoglobulin, Monoclonal antibody, Health facility, Bites and stings

## Abstract

**Background:**

Ensuring the uninterrupted availability of the anti-rabies vaccine (ARV) and rabies immunoglobulin (RIG) in health facilities is crucial to achieve the global target of zero dog-mediated human rabies deaths by 2030. This study aimed to estimate the availability of ARV and RIG across health facilities in India.

**Methods:**

We conducted a nationwide health facility-based, cross-sectional study across 60 districts selected by multistage probability sampling from 15 Indian states. In each district, we selected nine health facilities. We interviewed staff involved in the rabies control program in each of the selected health facilities, and abstracted and physically validated information on the availability of ARV and RIG.

**Findings:**

Of the 534 health facilities surveyed, 467 (87.5%) were public sector health facilities. ARV was available in 372 (79.7%, 95% CI: 75.7%–83.2%) public sector health facilities, ranging from 60.0% to 93.2% in different geographic regions. Availability of ARV was lowest in Urban Primary Healthcare Centres (UPHCs) (58.9%, 95% CI: 45.0%–71.9%). RIG was available in 95 (20.3%, 95% CI: 16.8%–24.3%) public sector health facilities, with the highest availability in southern states (27/88, 30.7%). The availability of RIG ranged from 1.8% (95% CI: 16.8%–24.3%) in UPHCs to 69.2% (95% CI: 48.2%–85.7%) in medical college hospitals.

**Interpretation:**

Considerable geographic and facility-level variations exist in the availability of ARV and RIG across India. Bridging the gap in the availability of ARV and RIG should be prioritised to achieve the goal of zero-dog-mediated human rabies deaths by 2030.

**Funding:**

10.13039/501100001411Indian Council of Medical Research.

**Research in context**

**Evidence before this study**

On January 29, 2025, we conducted a PubMed search using the key terms “rabies,” “animal bite,” “vaccine,” “immunoglobulin,” and “India.” We restricted our search to articles published in English from January 1, 2005, onwards. Our search identified seven research articles examining the procurement, distribution, and availability of anti-rabies vaccine and rabies immunoglobulin in India. Among these, two were qualitative studies based on key informant interviews with stakeholders involved in the country's rabies control program. Two studies were based on a multicentric, hospital-based cross-sectional survey conducted at 35 anti-rabies clinics across seven Indian states. While these studies highlighted gaps in the availability of rabies biologicals in different regions, none provided a comprehensive picture on the availability of these rabies biological in the country, especially the availability by geographic regions and health facility types.

**Added value of this Study**

In this study we did a nation-wide representative health facility survey spanning across all regions of India to describe the availability of anti-rabies vaccine and rabies immunoglobulin. We found that nearly four fifths of the public health facilities had anti-rabies vaccine. However, the availability was lowest in the north-east region and highest in the southern states. Two-third of the public facilities providing anti-rabies vaccine followed had adopted the dose saving intradermal regimen as recommended by the National Rabies Control Program, while the remaining were still following the older intra-muscular regimen. Regarding rabies immunoglobulin only one-fifth of public health facilities visited had it available. Nearly two-third of the medical college hospitals had rabies immunoglobulin available, but the availability was notably low in the public primary care facilities.

**Implications of all the available evidence**

Ensuring accessible and affordable post-exposure prophylaxis is a crucial strategy for achieving the goal of rabies elimination. Majority of the surveyed health facilities had anti-rabies vaccine available, but the eastern and north-eastern parts of India showed lower availability of the rabies biologicals. Bridging this distribution gap of anti-rabies biologicals are critical in achieving accessible and affordable post-exposure prophylaxis to all.

## Introduction

Rabies is an important global public health problem, causing around 59,000 human deaths annually, with Asia and African regions accounting for 95% of the global burden.^1^ Although rabies is 100% fatal once symptoms appear, it is preventable through the timely administration of a full course of post-exposure prophylaxis (PEP) after an animal bite. A recent nationwide survey estimated around 5700 dog-mediated human rabies deaths occurring annually in India.^2^

The global health community is committed to eliminating dog-mediated human rabies deaths by 2030. In 2015, the Tripartite alliance comprising of Food and Agriculture Organisation, World Organisation for Animal Health, and World Health Organisation issued a call for action to eliminate dog-mediated human rabies by 2030 at the Global Rabies Conference. United Against Rabies initiative is advancing this goal through the One Health approach.^3^ The zero by 30 strategy—the global plan to eliminate dog-mediated human rabies deaths by 2030 has instilled a sense of urgency in implementing control strategies. One core strategy towards achieving this goal is to ensure the availability of affordable post-exposure prophylaxis.^4^

In alignment with this global initiative, India launched the national action plan for dog mediated rabies elimination by 2030 under the national rabies control program (NRCP) to accelerate progress towards the 2030 target. A vital component of this plan is to ensure the availability of affordable rabies biologicals—anti-rabies vaccine (ARV) and rabies immunoglobulin (RIG) for all animal bite cases at every level of health care.^5^

In the past two decades, India has witnessed a 75% decline in dog-mediated human rabies deaths,^2^ most likely due to the increased availability of ARV and RIG in the health facilities. However, following the introduction of the national action plan, no comprehensive study has evaluated the availability and accessibility of ARV and RIG across the country. Understanding the availability of these biologicals by different geographic regions and by health facility types is crucial to guide the NRCP in focusing its efforts to ensure the availability ARV and RIG to the bite cases at the nearest health facility. The non-availability of ARV in the health facility on the day of the visit could lead to bite victims returning home without receiving ARV, which can undermine the efforts toward the goal of rabies elimination.^6^

With this background, we conducted a national-level health facility survey to estimate the availability of ARV and RIG across public and private health facilities in India.

## Methods

We conducted a nationwide health facility survey across 60 districts in India, selected using a multistage probability sampling, between March 2, 2022, and August 26, 2023. The survey was conducted alongside the community survey to estimate the burden of animal bites and human rabies deaths in the country. The districts selected for the health facility survey were the same as those selected for the community survey.

The methods for the community survey are described elsewhere.^2^ Briefly, we adopted a multistage cluster sampling approach. The country was stratified into five zones (north, south, east, west, and northeast). In the first stage, three states were selected by simple random sampling from each zone. In the second stage, four districts were selected within each state using probability proportionate to size method. The list of selected states and districts is provided in Appendix 1. In each selected district, we surveyed nine healthcare facilities closest to the clusters selected for the community survey. These included four primary care facilities (Primary Health Centres (PHCs) or Community Health Centres (CHCs)), two secondary care (Taluka, sub-district, or district hospitals) facilities, one tertiary care (medical college hospital or a government-owned multi/super-speciality hospital) facility from the public sector, and two private health facilities (private clinics or hospitals).

At each of these selected health facilities, we interviewed all staff directly involved in implementing the rabies control program, including medical doctors, staff nurses, and pharmacists. During the interview with the staff, we collected information on the procurement, storage, and administration of rabies biologicals, and staff training status about the rabies control program. The availability of ARV and RIG in the health facility was assessed by directly observing the facilities' stocks. We also conducted a review of ARV and RIG stock registers to verify the stock-out incidences of rabies biologicals in the health facility in the past one-year period. Document reviews were also performed, when conflicting responses were given by different staff members from the same facility.

In this study, a health facility was considered to have ARV or RIG available if at least one vial of the respective rabies biologicals was physically observed in the health facility at the time of the visit. A facility was considered to have an adequate stock of ARV for the next 15 days, if the current stock in the health facility was equal to or more than half the average monthly usage for the previous three months. A facility that reported at least one stock-out incidence in the past year was defined as any facility whose ARV stocks dropped to zero even for one day in the past one year. This was verified by observing the ARV stock register.

The data were collected electronically, using the Open Data Kit mobile application. We developed an interview guide and conducted detailed interviews with the district and state-level program officers involved in the rabies control program to inquire in detail about the vaccine procurement, distribution strategies and funding.

### Statistical analysis

We described the availability of ARV, RIG, and administration of ARV as per the intra-dermal (ID) updated thai red cross regimen by geographic zones and type of facilities as proportion with a 95% confidence interval. We summarised other health facility characteristics related to rabies control programs as proportions. All analyses were done using STATA v.17. R 4.4.1 was used for data visualization. The qualitative information was summarised in a predetermined format in Microsoft Excel.

### Ethical consideration

We received approval from the Institutional Human Ethics Committee of the Indian Council of Medical Research–National Institute of Epidemiology (NIE/IHEC/201910-01) and all participating institutions. We also got permission from the state and district health departments and the heads of health facilities for data abstraction for the health facility survey.

### Role of the funding source

The funder had no role in the study design, data collection, analysis, interpretation, and report writing.

## Results

We surveyed 534 health facilities across 60 districts from 15 Indian states. Of these, 467 (87.5%) were public sector health facilities, including 320 (60.0%) primary, 121 (12.7%) secondary, and 26 (4.9%) tertiary care institutions. (Appendix 2).

**Fig. 1:**
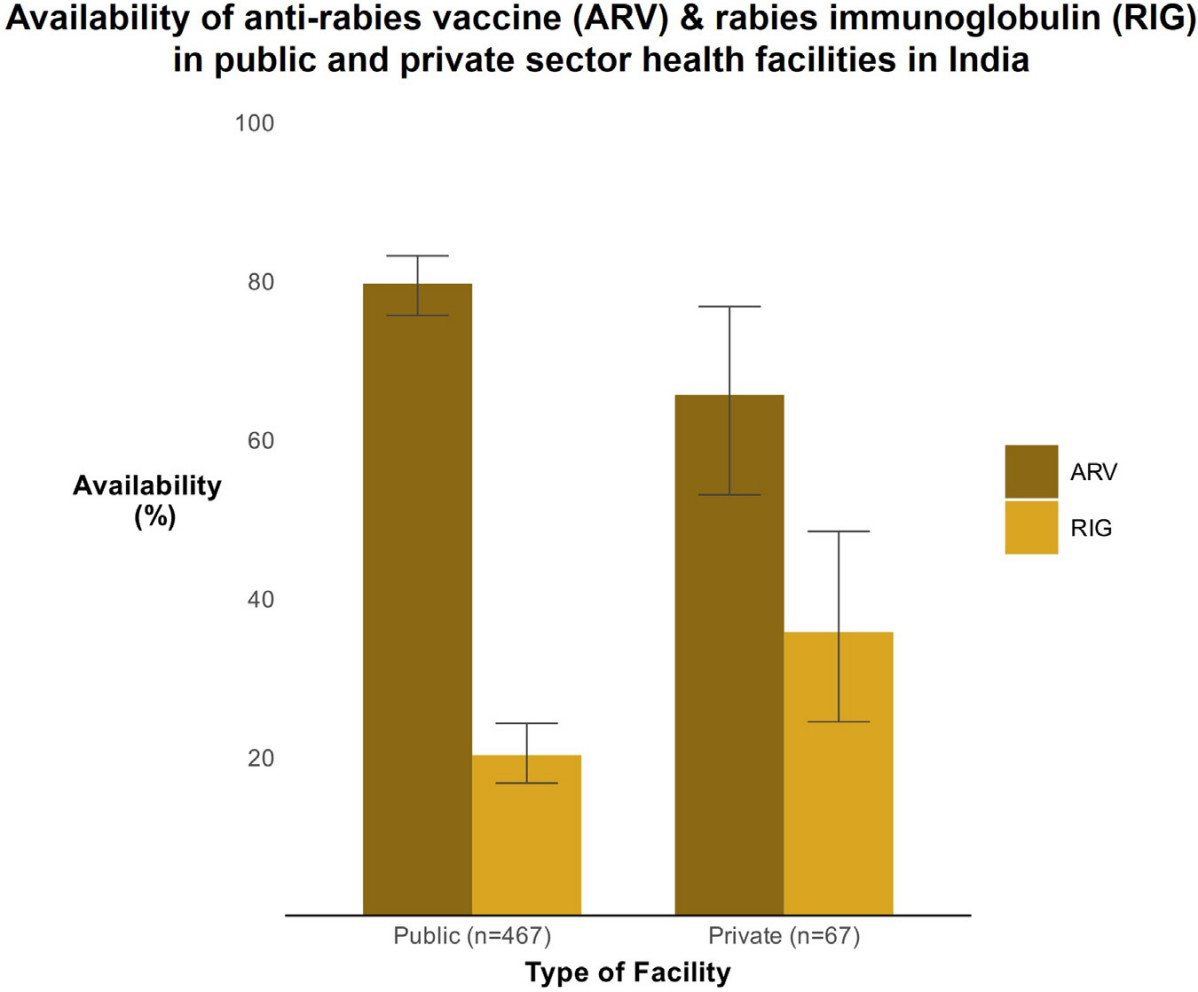
Availability (%) of anti-rabies vaccine (ARV) and rabies immunoglobulin (RIG) across public and private sector health facilities in India. Error bars represent 95% confidence intervals.

**Fig. 2:**
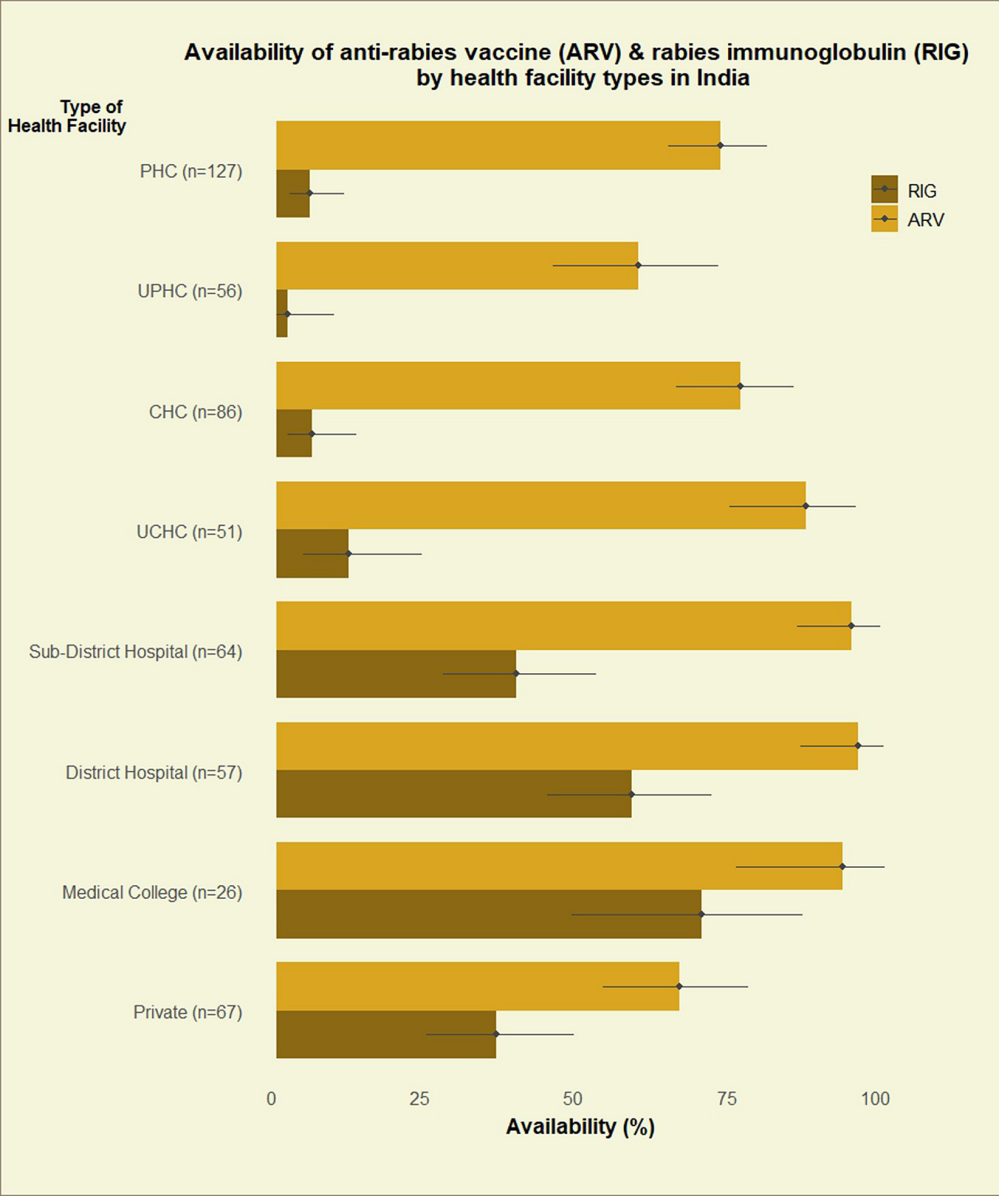
Availability (%) of anti-rabies vaccine (ARV) and rabies immunoglobulin (RIG) across different types of health facilities. Error bars represent 95% confidence intervals.

Among the 467 public sector health facilities, ARV was available in 372 (79.7%, 95% CI: 75.7–83.2) facilities (Fig. 1). ARV was available in more than 90% of the secondary and tertiary care facilities. The lowest availability (58.9%, 95% CI: 45.0–71.9, 33/56) was noted in urban PHCs and the highest availability was noted in district hospitals (94.7%, 95% CI: 85.4–98.9, 54/57) and medical college hospitals (92.3%, 95% CI: 74.9–99.0, 24/26) (Fig. 2).

**Fig. 3:**
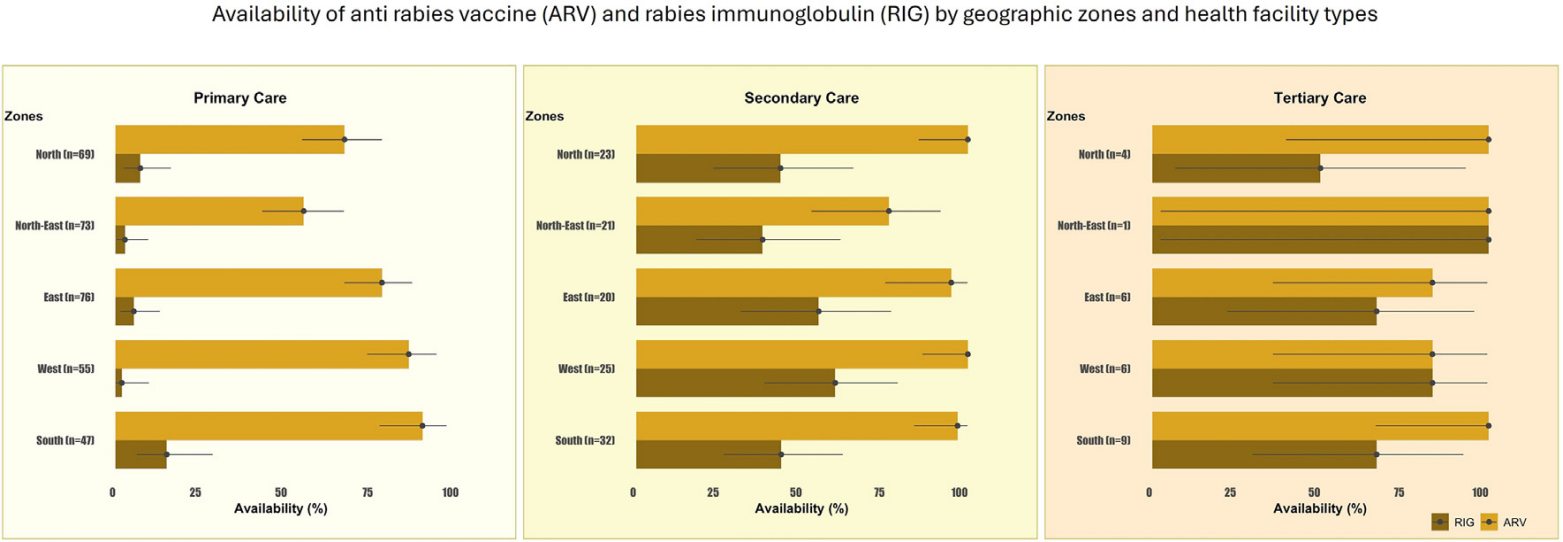
Availability (%) of Anti-Rabies Vaccine (ARV) and Rabies Immunoglobulin (RIG) across government health facilities by level of care and geographic zone in India. Error bars represent 95% confidence intervals.

Geographically, the availability of ARV was highest in the south zone (93.2%, 82/88), followed by the west (89.5%, 77/86), east (81.4%, 83/102) and north zones (76.0%, 73/96). The lowest availability of ARV was noted in the north-eastern states (60%, 57/95). The availability of ARV in primary care centres varied across geographic regions, while it remained consistently high in secondary and tertiary care centres (Fig. 3 & Appendix 3). Of the 372 health facilities with ARV, 349 (93.8%) had sufficient stock of ARV for the next 15 days on the day of visit, and 96 (25.8%) of the facilities reported at least one instance of complete stockout in the past year (Table 1).

**Table 1:** Health facility characteristics related to **a**nti-**r**abies **v**accine (ARV) administration, supply and storage among the public and private **sector** health facilities providing **ARV** in India**, human rabies health facility survey, 2022–23**.

Characteristics	Public (N = 372)	Private (N = 44)
	n (%, 95% CI)	n (%, 95% CI)
**Regimen followed for ARV administration**		
Updated Thai Red Cross Regimen – ID	224 (60.2, 55.0–65.2)	9 (20.5, 9.8–35.3)
Essen's Regimen - IM	124 (33.3, 28.6–38.4)	32 (72.7, 57.2–85.0)
Both	24 (6.5, 4.2–9.4)	3 (6.8, 1.4–18.7)
**ARV in the health facility**	n (%)	n (%)
ARV stock available for next 15 days on the day of visit	349 (93.8)	27 (61.4)
Reported ≥1 stockout previous year	96 (25.8)	6 (13.6)
**Preferred site for ARV**		
Deltoid	371 (99.7)	43 (97.7)
Anterolateral thigh	–	1 (2.3)
Gluteus	1 (0.3)	–
**ARV administered by**	n (%)	n (%)
Doctor	40 (10.8)	16 (36.4)
Nurse	298 (80.1)	26 (59.1)
Others (Pharmacist)	34 (9.1)	2 (4.5)
**ARV storage**		
Refrigerator (2 °C–8 °C)	341 (91.7)	43 (97.7)
ILR (2 °C–8 °C)	31 (8.3)	1 (2.3)
**Method adopted for procurement of ARV**		
Centralized purchase from the state	359 (96.5)	–
Local purchase through tender	10 (2.7)	5 (11.4)
Local purchase without tender	3 (0.8)	39 (88.6)

Of the 44 private health facilities providing ARV, 72.7% (n = 32) followed the intramuscular (IM) essen's regimen. In both public and private health facilities, ARV was most commonly administered by the staff nurse. Deltoid was the most preferred site for vaccine administration in both ID and IM regimens (Table 1).

The ID updated thai red cross regimen given on day 0-3-7-28 (2-2-2-0-2) was followed by 224 (60.2%, 95% CI: 55.0–65.2) public sector health facilities providing ARV. The ARV administration using ID regimen was lowest (56.5%, 133/234) in primary care centres. More than 70% of the health facilities in the north, south, and west regions followed the ID regimen. However, less than 40% of the facilities in the northeast and east zones followed the ID regimen (Appendix 4).

Of the 467 public sector health facilities, RIG was available in 95 health facilities (20.3%, 95% CI: 16.8, 24.3). The availability varied by the type of health facilities—5.9% (19/320) in primary, 47.9% (58/121) in secondary, and 69.2% (18/26) in tertiary care facilities. Only 1.8% (1/56) of urban PHCs had RIG (Fig. 2 & Appendix 5). More than half of the private health facilities (24/44, 54.5%) providing ARV also had rabies immunoglobulin.

Most of the public sector health facilities (n = 75, 78.9%) were using equine rabies immunoglobulin (ERIG) for passive immunisation of category III wounds. On the other hand, half (50.0%, 12/24) of the private health facilities used human rabies immunoglobulin (HRIG) or rabies monoclonal antibodies (RmAb). All facilities were practising the technique of infiltrating into the wound to administer RIG. In private and public facilities, the medical doctors primarily administered RIG. 55 (73.3%) of the public and 8 (88.9%) of the private health facilities providing (ERIG) performed allergic skin sensitivity testing before administration of the RIG (Table 2).

**Table 2:** Health facility characteristics related to **r**abies **i**mmunoglobulin (RIG) administration in public and private **sector** health facilities in India**, human rabies health facility survey, 2022–23**.

Characteristics	Public **(N = 95)**	Private **(N = 44)**
	n (%)	n (%)
**Type of RIG provided**^a^		
Human rabies immunoglobulin (HRIG)	23 (24.2)	12 (50.0)
Equine rabies immunoglobulin (ERIG)	75 (78.9)	9 (37.5)
Rabies monoclonal antibodies (RmAb)	3 (3.2)	13 (54.2)
**Preferred site for RIG administration**		
Infiltrate into the wound	95 (100)	24 (100)
**Utilization of leftover RIG after infiltrating into the wound**		
Give as intra-muscular	80 (84.2)	20 (83.3)
Inject around the wound itself	11 (11.6)	3 (12.5)
Not given	4 (4.2)	1 (4.2)
**RIG administered by**		
Doctor	50 (52.6)	15 (62.5)
Nurse	39 (41.1)	9 (37.5)
Pharmacist	6 (6.3)	–
	N = 75	N = 9
**Performed skin sensitivity test before ERIG administration**^b^	55 (73.3)	8 (88.9)

a Only the health facilities providing RIG are included.

b Only facilities providing ERIG are included.

Health facilities providing ARV followed two approaches to inform the vaccination schedule with the bite cases. The first approach was to share the schedule by writing it on the out-patient prescription, and the other was to distribute specially designed ARV vaccination cards. ARV vaccination cards were provided only in 89 (23.9%) public and 18 (40.9%) private sector health facilities. Fewer than one-third (31.2%, 116/372) of public sector health facilities actively followed up with the bite cases for reminders regarding the subsequent doses. The bite cases were contacted either by telephone or were tracked with the help of the field health workers such as ASHA (Accredited Social Health Activists). In most facilities, the medical doctors categorised the wound and had soap and functional water taps for wound washing. Fewer than half (45.4%, 169/372) of the government facilities had at least one staff member recently trained under the rabies control programme (Table 3).

**Table 3:** Health facility characteristics related to the rabies control program among the health facilities providing Anti-Rabies Vaccine (ARV) in India**, human rabies health facility survey, 2022–23**.

Characteristics	Public (N = 372)	Private (N = 44)
	n (%)	n (%)
**Facilities receiving animal bite cases**		
IEC (Information Education Communication) material on animal bite management displayed in the facility campus	97 (26.1)	1 (2.3)
Provided ARV vaccination card	89 (23.9)	18 (40.9)
Animal bite reporting system	333 (89.5)	4 (9.1)
Facilities with at least one staff recently trained/oriented in rabies control programme (wound management, wound classification, ARV-ID administration, reporting etc.)	169 (45.4)	6 (13.6)
Availability of animal bite register	353 (94.9)	5 (11.4)
Side effects reported after vaccination	13 (3.5)	1 (2.3)
**Patient followed up for completion of vaccination schedule (if patients do not report for subsequent doses)**		
Inform health worker	49 (13.2)	–
Telephonic follow-up	67 (18.0)	6 (13.6)
Do not follow-up	256 (68.8)	38 (86.4)
**Wound categorization done by**		
Doctor	352 (94.6)	43 (97.7)
Nurse	13 (3.5)	–
Categorized by others	7 (1.9)	1 (2.3)
**Wound management**^a^	N = 343	N = 44
Wound washing available inside the facility^b^	278 (81.1)	31 (70.5)
**Human rabies case management**^c^	N = 147	N = 44
Treating rabies cases in the facility	14 (9.5)	3 (6.8)
Have specialized ward for treating rabies cases	11 (7.5)	2 (4.5)
Maintain line list of cases treated for rabies	4 (2.7)	–

a Only health facilities providing ARV excluding those in Tamil Nadu were considered.

b All facilities with functional tap(s) with water and soap are considered provisions for wound washing available.

c Primary care centers (PHC/UPHC/CHC/UCHCs) are not considered for the indicator.

ARV and RIG are provided free of cost in most of the public sector health facilities in India. States implemented their own mechanisms for procuring and distributing ARV and RIG. The National Health Mission (NHM) was the primary funding source for vaccine procurement. In cases of funding shortages, additional funds were sourced from the state health budget, district funds, local body, or health facility funds. Each state designated a nodal institute or corporation to coordinate the procurement and supply of biologicals. The state health department or directorate transferred funds to the nodal agency, which coordinated the procurement procedure through a competitive bidding process and managed the distribution of rabies biologicals. Though the overall process for procurement and distribution of ARV and RIG was broadly similar across states, variations existed in the choice of nodal agency, timelines for fund transfers, procurement procedures, vaccine stock intending mechanism, and the role of district-level institutions in distribution.

## Discussion

Our study highlights a considerable variability in the availability of Anti-Rabies Vaccine and Rabies Immunoglobulin across different levels of healthcare facilities and geographic regions in India. While ARV was available in approximately 80% of public health institutions, there were disparities between primary, secondary, and tertiary care levels. The availability of both ARV and RIG was the lowest in the primary care health facilities. Similiar disparity was also evident in the availability of ARV and RIG across different geographic regions, especially in the northeast, north and eastern zones.

Primary healthcare facilities are the first point of contact for the public to the health system.^7^ Ensuring the availability of ARV and RIG at all primary healthcare facilities at zero cost can help to increase coverage and compliance to PEP.^8^, ^9^, ^10^ A recent study, which assessed the availability of ARV and RIG in public sector health facilities across 23 countries in Asia and Africa, found that the geographical access to ARV and RIG was limited, especially in the primary care health facilities in most of these countries.^11^^,^^12^ The variation in the availability of rabies biologicals between geographic zones and health facility types can be influenced by a complex interplay of multiple societal, market, and system-level factors. These may include, but are not limited to, factors like differences in funding transfer mechanisms, limited recognition of rabies as a public health priority, operational challenges in procurement and supply chain processes and fragmented stakeholder coordination.^11^^,^^13^

In India, two regimens are followed for anti-rabies vaccination: the IM Essen's regimen (1-1-1-1-1) and the ID Updated Thai Red Cross regimen (2-2-2-0-2). The ID administration of the anti-rabies vaccine is dose-saving and equally efficacious as the IM injection.^14^^,^^15^ Since 2006, India has adopted this universal switch to the ID regimen for PEP. Our survey showed that nearly two-thirds of government health facilities across India have shifted to the ID route for ARV administration. This is an important achievement considering the fact that many countries in Asia are still following the IM Essen's Regimen.^11^ However, the fact that only close to one-third of the facilities in the east and northeast regions have adopted the ID route for ARV administration indicates a gap in the program implementation.

Evidence suggests that a universal switch to an ID regimen can reduce the financial burden of anti-rabies vaccination by up to 60%.^16^^,^^17^ The use of the new abridged Cambodia regimen recommended by WHO in its 2018 position paper^18^ can further increase vaccine equity by allowing 33% more patients to be treated with the available doses while also reducing the direct costs of vaccination, transportation, and other indirect costs to the vaccine recipients.^19^^,^^20^

However, intradermal vaccine administration has some operational challenges. Mainly, the need for trained healthcare workers to correctly administer ARV and availability of intradermal syringes with fixed needles to prevent leakage.^21^ This is important as improper administration techniques can lead to vaccine failure.^22^ Identifying and addressing these implementation challenges will enhance the affordability and accessibility of PEP to the bite cases, and can help in the reduction of the human rabies deaths.^23^

Rabies immunoglobulins are crucial for managing category III animal bites.^5^^,^^18^ However, our study revealed that RIGs were available in only one in five public sector health facilities. Availability of RIG in secondary and tertiary care facilities ranged between 40% and 70%. These findings align with those of Sudarshan et al. (2019) and Hanumanthaiah et al. (2019).^24^^,^^25^ Studies from selected countries in Africa and Asia have also reported that the availability of RIG was limited to only the important cities, and sporadic or irregular supply was noticed in some of the smaller towns, semi-urban and rural places in these countries.^11^^,^^12^^,^^26^ NRCP guidelines recommend that any wound with bleeding should be treated with RIG in addition to ARV. In practice, over half of all animal bite wounds reported to health facilities are likely to be categorised as category III, requiring RIG as part of PEP.^27^ The scarcity of RIG in primary care facilities can lead to bite cases being referred to higher centres, resulting in increased vaccination costs for individuals, higher case-loads at advanced centres, or even the omission of RIG in PEP.^28^ This situation underscores the necessity of making RIG available at primary health care centres alongside ARV.

Ensuring the availability of RIG in all health facilities across India requires an increase in the production of RIG. In this context, RmAb presents as a promising alternative to both HRIG and ERIG. Compared to ERIG, RmAb offers the potential scope for large-scale production with standardised quality, elimination of animal use in the manufacturing, and reduced risk of adverse events.^18^^,^^29^ Also, it is cheaper compared to HRIG.^30^ Studies have shown that RmAbs are safe for human use, but the number of studies on their efficacy and safety in the Indian context are limited.^31^ This highlights the need for larger clinical trials and post-marketing surveillance to assess the safety and efficacy of rabies monoclonal antibodies in the Indian setting.^32^

Evidence from countries like Tanzania and Thailand showed that improved access to affordable PEP can help reduce human rabies deaths in the country.^9^^,^^17^^,^^33^ Our study shows India performs better in providing accessible and affordable PEP to the people through public sector health facilities compared to many countries in the Asian and African regions.^11^^,^^26^ However, variability in the PEP provision by geographic regions and type of health facilities exists as an implementation challenge in the program that requires attention. Addressing this requires an understanding of the barriers that lead to the limited availability of rabies biologicals in the local geographical settings. Based on these findings, context-specific strategies need to be developed to improve the supply chain for rabies biologicals. The decentralised approach adopted for the procurement and distribution of rabies biologicals in India provides states the flexibility to implement tailored solutions regionally to maintain an uninterrupted supply of ARV and RIG at all primary care health facilities.

Our study had certain limitations. Health facilities for the survey were not selected randomly and hence may introduce selection bias, potentially limiting the generalizability of the results. However, this health facility survey was conducted alongside a community survey. For the community survey, multistage probability sampling was used to select clusters. We selected facilities closest to these clusters for the health facility survey, reducing investigator bias in the selection process. Additionally, our findings are based on observations made during visits to health facilities and responses from their staff, potentially leading to social desirability bias. To mitigate this, our data collectors were trained to verify staff responses with hospital records and through cross-questioning.

### Conclusion

In India, the Anti-Rabies Vaccine was available in 80% of public sector health facilities, while Rabies Immunoglobulin was available in about 20% of public sector health facilities. There was a considerable disparity in the distribution of ARV and RIG across different geographical regions and types of health facilities. Achieving the goal of zero dog-mediated human rabies deaths by 2030 requires addressing this gap in distribution equity. Nearly two-thirds of health facilities surveyed had switched to the cost-saving intradermal route for vaccine administration. Expanding this initiative to all health facilities in the country would improve vaccine equity by allowing more people to be treated with the available doses while also reducing the direct costs of vaccination for both the public and the government.

## Contributors

NSK, JWVT and MVM did the literature search.

NSK, JWVT and DSR did the study design.

NSK, JWVT, SD, AS, SE, DSR, SAK, AD, AKS, AM, BS, CP, DG, MM, NV, PS, RKS, SR, SMK, SB, TR, VVY, AkS, AsS, BVT, CD, DRP, KJ, MMR, MT, NVS, PG, SV, ShD, SKP,PV, AA, VGB, DKS, GPS, HM, KZ, KP, MPC, RK, RAV, SP, SA, SS, VBM, RSV, AV, SJ, MR, MVM did the data collection.

NSK, JWVT SD, AS, SE, CP, SR and MVM did the data analysis.

JNSK, JWVT, SD, AS, SE, DSR, SAK, AD, AKS, AM, BS, CP, DG, MM, NV, PS, RKS, SR, SMK, SB, TR, VVY, AkS, AsS, BVT, CD, DRP, KJ, MMR, MT, NVS, PG, SV, ShD, SKP, PV, AA, VGB, DKS, GPS, HM, KZ, KP, MPC, RK, RAV, SP, SA, SS, VBM, RSV, AV, SJ, MR, MVM did the project management.

NSK, JWVT, SD, AS, SE, DSR and MVM did the data interpretation.

NSK, JWVT, SD, AS, SE, DSR and MVM accessed and verified the data.

NSK, JWVT and MVM wrote the first draft of the manuscript.

All authors approved the final draft of the manuscript.

## Data sharing statement

All key anonymised data collected during the study, along with a data dictionary are available upon request to the corresponding author, after approval of a proposal with a signed data access agreement.

## Disclosure of AI

While preparing this manuscript, we used Grammarly AI and ChatGPT to improve readability and language. After using this tool, we reviewed and edited the content as needed and take full responsibility for the content of the publication.

## Editor note

The Lancet Group takes a neutral position with respect to territorial claims in published maps and institutional affiliations.

## Declaration of interests

We declare no competing interests.
