# Supplementary material for: Availability of anti-rabies vaccine and rabies immunoglobulin in Indian health facilities: a nationwide cross-sectional health facility survey
Source: Lancet Reg Health Southeast Asia. 2025 Jun 13;38:100608. doi: 10.1016/j.lansea.2025.100608 (PMC12205764; doi:10.1016/j.lansea.2025.100608)
Supplement: Supplementary File [file mmc1.docx]

**Appendix 1. States and district selected for health facility survey**

**Appendix 2. Health facilities visited by geographic zones and type of health facilities during the health facility survey**

**Appendix 3: Availability of ARV by zone and by type of health facilities health facilities providing Anti-Rabies Vaccine in India**

**Appendix 4: Coverage of the Intra-dermal (ID) Updated Thai Red-cross regimen by zone and type of health facilities among health facilities providing Anti-Rabies Vaccine in India**

**Appendix 5: Availability of RIG availability by zone and by type of health facilities among health facilities in India**

**Appendix 1. States and district selected for health facility survey**


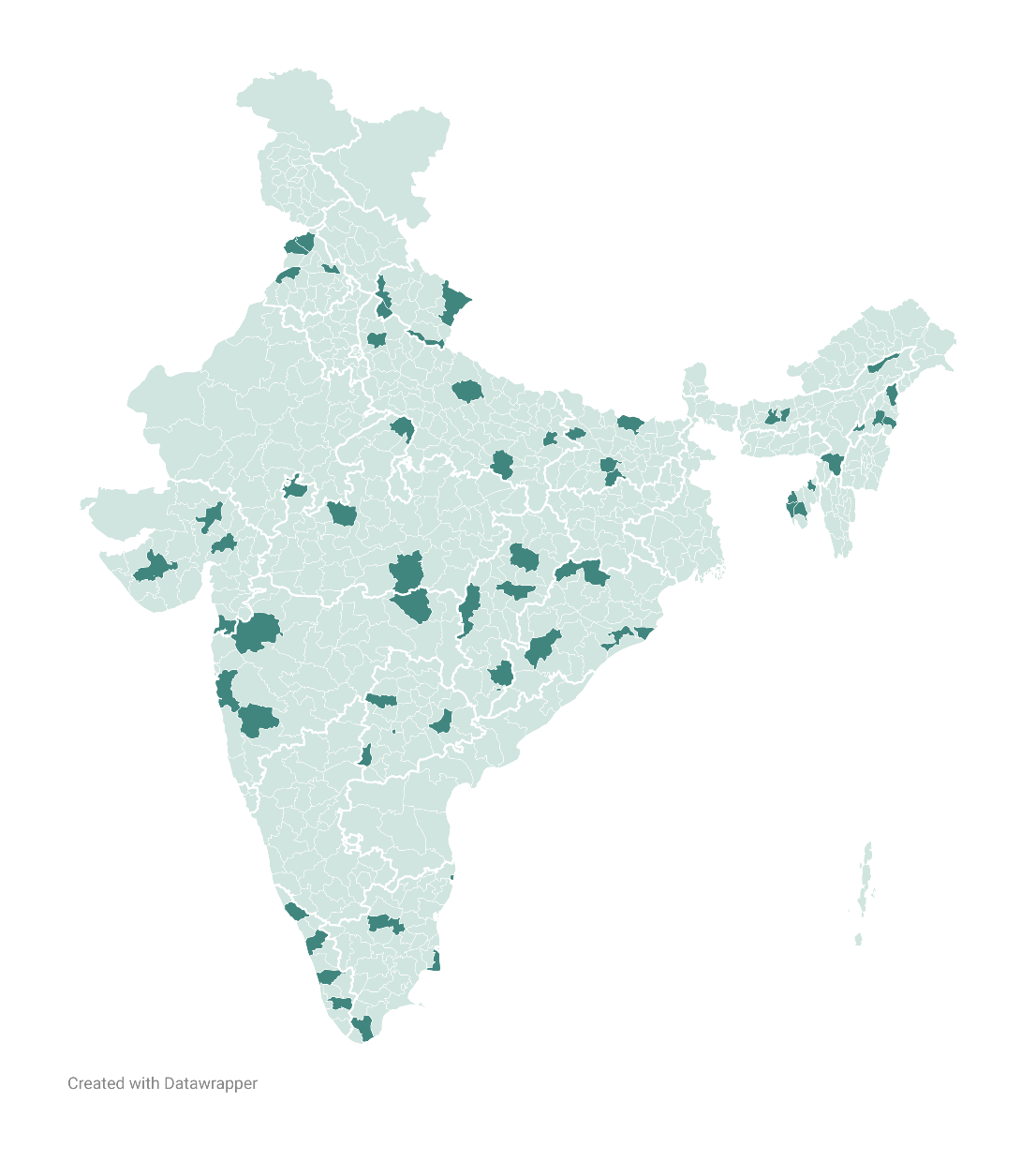

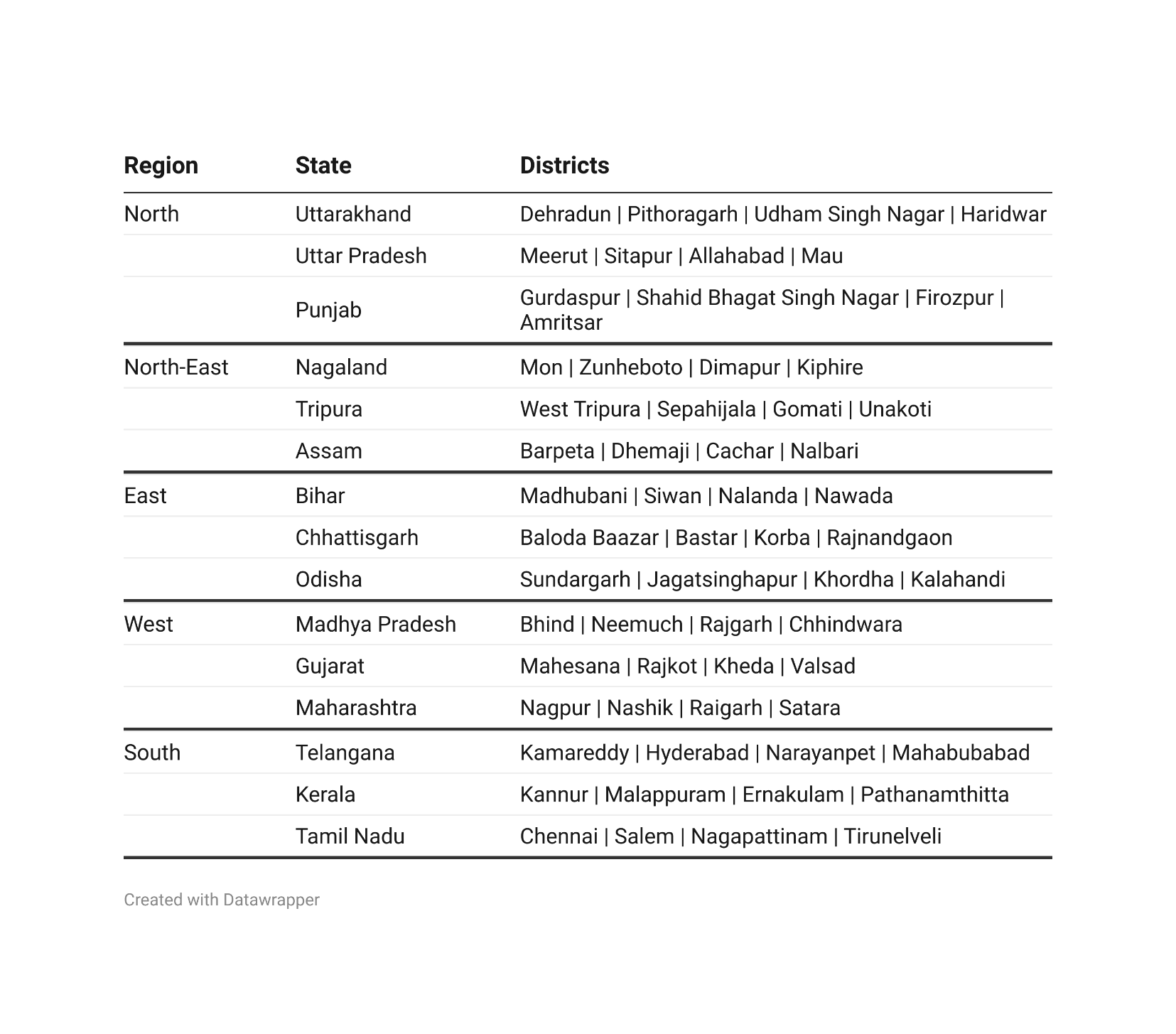


These districts selected for health facility survey are represented as dark green shaded in the map

**Appendix 2. Health facilities visited by geographic zones and type of health facilities during the health facility survey**

| Facilities | North | North-east | East | West | South | Total |
| --- | --- | --- | --- | --- | --- | --- |
| PHC | 18 | 35 | 30 | 26 | 18 | 127 |
| Urban PHC | 6 | 6 | 13 | 14 | 17 | 56 |
| CHC | 29 | 22 | 24 | 7 | 4 | 86 |
| Urban CHC | 16 | 10 | 9 | 8 | 8 | 51 |
| Sub-District Hospital | 13 | 10 | 10 | 16 | 15 | 64 |
| District Hospital | 10 | 11 | 10 | 9 | 17 | 57 |
| Medical College | 4 | 1 | 6 | 6 | 9 | 26 |
| Overall Public | 96 | 95 | 102 | 86 | 88 | 467 |
| Private | 14 | 7 | 11 | 20 | 15 | 67 |
| Total facilities by regions | 110 | 102 | 113 | 106 | 103 | 534 |

**Appendix 3: Availability of ARV by zone and by type of health facilities health facilities providing Anti-Rabies Vaccine in India**

| **Type of facility** | **North** | | | **North-East** | | | **East** | | | **West** | | | **South** | | |
| --- | --- | --- | --- | --- | --- | --- | --- | --- | --- | --- | --- | --- | --- | --- | --- |
|  | N | n | % | N | n | % | N | n | % | N | n | % | N | n | % |
| PHC | 18 | 1 | 5.6 | 35 | 29 | 82.9 | 30 | 22 | 73.3 | 26 | 25 | 96.2 | 18 | 15 | 83.3 |
| UPHC | 6 | 0 | 0 | 6 | 2 | 33.3 | 13 | 8 | 61.5 | 14 | 7 | 50.0 | 17 | 16 | 94.1 |
| CHC | 29 | 29 | 100 | 22 | 5 | 22.7 | 24 | 21 | 87.5 | 7 | 7 | 100 | 4 | 3 | 75.0 |
| UCHC | 16 | 16 | 100 | 10 | 4 | 40.0 | 9 | 8 | 88.9 | 8 | 8 | 100 | 8 | 8 | 100 |
| SDH | 13 | 13 | 100 | 10 | 8 | 80.0 | 10 | 9 | 90.0 | 16 | 16 | 100 | 15 | 14 | 93.3 |
| DH | 10 | 10 | 100 | 11 | 8 | 72.7 | 10 | 10 | 100 | 9 | 9 | 100 | 17 | 17 | 100 |
| MCH | 4 | 4 | 100 | 1 | 1 | 100 | 6 | 5 | 83.3 | 6 | 5 | 83.3 | 9 | 9 | 100 |
| Private | 14 | 10 | 71.4 | 7 | 6 | 85.7 | 11 | 9 | 81.8 | 20 | 12 | 60.0 | 15 | 7 | 46.7 |

**Appendix 4: Coverage of the Intra-dermal (ID) Updated Thai Red-cross regimen by zone and type of health facilities among health facilities providing Anti-Rabies Vaccine in India**

| **Type of facility** | **North** | | | **North-East** | | | **East** | | | **West** | | | **South** | | |
| --- | --- | --- | --- | --- | --- | --- | --- | --- | --- | --- | --- | --- | --- | --- | --- |
|  | N | n | % | N | n | % | N | n | % | N | n | % | N | n | % |
| PHC | 18 | 0 | 0 | 35 | 2 | 5.7 | 30 | 0 | 0 | 25 | 0 | 0 | 18 | 5 | 27.8 |
| UPHC | 6 | 0 | 0 | 6 | 0 | 0 | 13 | 0 | 0 | 14 | 0 | 0 | 17 | 1 | 5.9 |
| CHC | 29 | 3 | 10.3 | 22 | 0 | 0 | 24 | 1 | 4.2 | 7 | 1 | 14.3 | 4 | 0 | 0 |
| UCHC | 16 | 2 | 12.5 | 10 | 0 | 0 | 9 | 3 | 33.3 | 8 | 0 | 0 | 8 | 1 | 12.5 |
| SDH | 13 | 6 | 46.2 | 10 | 4 | 40.0 | 10 | 5 | 50.0 | 16 | 6 | 37.5 | 15 | 4 | 26.7 |
| DH | 10 | 4 | 40.0 | 11 | 4 | 36.4 | 10 | 6 | 60.0 | 9 | 9 | 100 | 17 | 10 | 58.8 |
| MCH | 4 | 2 | 50.0 | 1 | 1 | 100 | 6 | 4 | 66.7 | 6 | 5 | 83.3 | 9 | 6 | 66.7 |
| Private | 14 | 2 | 14.3 | 7 | 1 | 14.3 | 11 | 7 | 63.6 | 20 | 8 | 40.0 | 15 | 6 | 40.0 |

**Appendix 5: Availability of RIG availability by zone and by type of health facilities among health facilities in India**

| **Type of facility** | **North** | | | **North-East** | | | **East** | | | **West** | | | **South** | | |
| --- | --- | --- | --- | --- | --- | --- | --- | --- | --- | --- | --- | --- | --- | --- | --- |
|  | N | n | % | N | n | % | N | n | % | N | n | % | N | n | % |
| PHC | 1 | 0 | 0 | 29 | 8 | 27.6 | 22 | 5 | 22.7 | 28 | 18 | 64.3 | 15 | 9 | 60.0 |
| UPHC | 0 | 0 | 0 | 2 | 0 | 0 | 8 | 1 | 12.5 | 7 | 5 | 71.4 | 16 | 9 | 56.3 |
| CHC | 29 | 25 | 86.2 | 5 | 4 | 80.0 | 21 | 8 | 38.1 | 7 | 6 | 85.7 | 3 | 3 | 100 |
| UCHC | 16 | 12 | 75.0 | 4 | 2 | 50.0 | 8 | 3 | 37.5 | 8 | 7 | 87.5 | 8 | 8 | 100 |
| SDH | 13 | 8 | 61.5 | 8 | 2 | 25.0 | 9 | 4 | 44.4 | 16 | 16 | 100 | 14 | 11 | 78.6 |
| DH | 10 | 7 | 70.0 | 8 | 4 | 50.0 | 10 | 5 | 50.0 | 9 | 8 | 88.9 | 17 | 12 | 70.6 |
| MCH | 4 | 3 | 75.0 | 1 | 0 | 0 | 5 | 0 | 0 | 5 | 5 | 100 | 9 | 6 | 66.7 |
| Private | 10 | 5 | 50.0 | 6 | 0 | 0 | 9 | 0 | 0 | 12 | 2 | 16.7 | 7 | 2 | 28.6 |
